# Complete genome sequence of *Exiguobacterium profundum* TSS-3 isolated from an extremely saline-alkaline spring located in Ixtapa, Chiapas-México

**DOI:** 10.1128/MRA.00171-23

**Published:** 2023-08-22

**Authors:** Reiner Rincón-Rosales, Marco A. Rogel, Clara Ivette Rincón-Molina, Gabriela Guerrero, Luis Alberto Manzano-Gómez, Aline López-López, Francisco A. Rincón Molina, Esperanza Martínez-Romero

**Affiliations:** 1 Departamento de Ingeniería Química y Bioquímica, Tecnológico Nacional de México / Instituto Tecnológico de Tuxtla Gutiérrez, Tuxtla Gutiérrez, Chiapas, Mexico; 2 Centro de Ciencias Genómicas, Universidad Nacional Autónoma de México, Cuernavaca, Morelos, Mexico; 3 Departamento de Investigación y Desarrollo, 3R Biotec SA de CV., Tuxtla Gutiérrez, Chiapas, Mexico; University of Southern California, Los Angeles, California, USA

**Keywords:** plant growth-promoting bacteria, *Exiguobacterium profundum*, NaCl resistant

## Abstract

We report the complete genome sequence of *Exiguobacterium profundum* TSS-3, a strain isolated from the sediment of an extremely saline-alkaline spring located in Ixtapa, Chiapas-México (16° 47´ LN and 92° 54´ LO). Its genome is composed of a 2.8-Mb chromosome and a small 4.6-Kb plasmid.

## ANNOUNCEMENT

*Exiguobacterium* strains have been isolated from different environments such as glaciers, seawater, soils, hydrothermal vents, industrial affluents, salt lakes, and rhizosphere plants (1, 2). Many strains in this genus have characteristics with potential for use in agriculture, industry, and bioremediation (3).

The spring sediment was collected in 2018 with sterilized 3L Van Dorn bottles (https://vimeo.com/365895946) at a depth of 1 m from the saltwater in a 17-m-deep well, following the Mexican standard NOM-021-RECNAT-2000 (4). One gram of sediment was resuspended in 50 mL of sterile water; it was diluted up to 10^−6^ and plated in lysogeny broth (LB) medium (5) to obtain isolated colonies. A colony designated TSS-3 was streak purified twice and stored at −70°C in LB containing 30% (vol/vol) glycerol. A glycerol-recovered colony was used to inoculate 5 mL of liquid LB; it was incubated overnight at 30°C for DNA extraction. The DNA was obtained with the “DNA Isolation Kit for Cells and Tissues” from Roche, following the company’s instructions. Libraries were prepared with “TruSeq Nano DNA, read length 2 × 151 bp” and “SQK-LSK109” Kits for the Novaseq 6000 platform and Oxford Nanopore Technologies, respectively. Different aliquots of the same DNA were used for sequencing with both methods. No size was selected for Nanopore; only a large fragment buffer was used in the final library wash. The flow cell chemistry for Nanopore was R9.4.1, and the basecaller used was Guppy v6.4.6+ae70e8f. The Nanopore N50 read length was 21,898 bp. A pre-assembly read QC was made with Trim Galore v2.5 for Illumina sequences (https://www.bioinformatics.babraham.ac.uk/projects/trim_galore/). Unicycler hybrid assembler v0.4.8 was used to get the complete and closed genome, with 24, 269,912 and 64,387 reads for Illumina and Nanopore, respectively (6). Genome coverage was 420×. Genome annotation was performed using the NCBI Prokaryotic Genome Annotation Pipeline Gene (7). Clusters of Orthologous Groups were obtained using CD-Search (Conserved Domain Search Service) from the NCBI database, and hits were accepted with an *E*-value ≤1e−10^6^. Default parameters were used for all software unless otherwise specified.

The TSS-3 strain has a 2.8-Mb chromosome and a small 4.6-Kb plasmid, which were confirmed by alkaline lysis (8). The predicted CDS were 2,900 and 6, with a GC content of 48.16% and 43.9% for the chromosome and small plasmid, respectively.

The species was confirmed with an ANI value of 96% against *Exiguobacterium profundum* type strain 10C^T^ with the accession number ASM2523463v1 (9, 10). *Exiguobacterium* reference genomes were downloaded from the NCBI database, and the PYANI v0.2.12 program was used to calculate the ANI value with the option “ANIm (ANImummer)” (11).

Genes annotated as cardiolipin synthase and the seven-gene operon involved in the Na+/H+ antiporter function could help strain TSS-3 to grow in extreme salinity conditions since it has been reported that some strains require them to grow in saline environments (12 - 15). The *E. profundum* TSS-3 genome shows different genes such as proteases, amylases, pullulanases, and neopullulunases that could have a wide application in industry.

## Data Availability

The whole-genome nucleotide sequence of *E. profundum* TSS-3 has been deposited in GenBank under the accession numbers CP109617, CP109618, and SRA: PRJNA887767.
